# Comparative effectiveness of kilo- and megavoltage energies in low-dose radiotherapy for painful degenerative musculoskeletal diseases: a systematic review and meta-analysis

**DOI:** 10.1007/s00066-024-02329-0

**Published:** 2024-12-04

**Authors:** Aram Kim, Jeanny Kwon, Ji Young Kim, Byoung Hyuck Kim

**Affiliations:** 1 Department of Radiation Oncology, Chungnam National University School of Medicine, Chungnam National University Hospital, Daejeon, Korea (Republic of); 2 Radiation Effects Research Section, Radiation Health Institute, Korea Hydro & Nuclear Power Co. Ltd, Seoul, Korea (Republic of); 3 Department of Radiation Oncology, Seoul National University College of Medicine, SMG-SNU Boramae Medical Center, 20, Boramae-ro 5-gil, Dongjak-gu, 07061 Seoul, Korea (Republic of)

**Keywords:** Osteoarthritis, Pain management, Radiotherapy, Low-dose, Chronic pain, Joint diseases

## Abstract

**Purpose:**

This study aimed to assess the impact of different energy levels on the effectiveness of low-dose radiotherapy (LDRT) for treating painful degenerative musculoskeletal diseases, as comparative efficacy data are currently lacking.

**Methods:**

A systematic review was conducted in PubMed, Embase, and the Cochrane Library databases to identify studies with response information on the energy used (kilovoltage [kV] vs. megavoltage [MV]). The primary endpoint was the overall response rate (ORR), and the secondary endpoint was the complete response rate (CRR). Exploratory subgroup analyses included treatment site, study period, study design, country, and dose per fraction.

**Results:**

A total of 33 studies involving 12,143 patients were analyzed. Short-term follow-up (up to 6 months) showed a pooled ORR of 64% (95% CI 46–78%) for kV and of 62% (95% CI 54–70%) for MV. Long-term follow-up (at least 12 months) revealed a pooled ORR of 85% (95% CI 65–95%) for kV and of 69% (95% CI 62–75%) for MV. Subgroup analysis indicated no significant differences in ORR for energy level stratified by treatment site and other factors. Regarding dose per fraction (0.5 Gy vs. 1.0 Gy), comparable ORRs were demonstrated between the two energies. No clinical side effects were noted.

**Conclusion:**

This meta-analysis suggests that the known effectiveness of LDRT in painful degenerative musculoskeletal disease may not depend on the energy used. Additional studies using standardized evaluation methods are warranted to establish consistency and enhance the comprehensiveness of research. Further research is also needed to explore treatment modality selection considering disease-specific biology.

**Supplementary Information:**

The online version of this article (10.1007/s00066-024-02329-0) contains supplementary material, which is available to authorized users.

## Introduction

Radiation therapy is usually employed for treating malignant diseases but has also been employed for treatment of benign painful musculoskeletal diseases (MSD) including osteoarthritis (OA), which is the most common degenerative joint disease [1]. There are numerous studies showing that low-dose radiotherapy (LDRT) has durable clinical effects in the treatment of painful musculoskeletal diseases without side effects [2, 3]. Not only the applied techniques of LDRT but also parameters including total dose, dose per fraction, target volumes, and energy spectrum differ among the various studies and have continuously been optimized during recent decades; disease-specific standard use of LDRT for the treatment of MSD is not well established.

When considering LDRT for MSD, both kilovoltage (kV; also known as orthovoltage) and megavoltage (MV) radiation machines can be used, but they have distinct differences in their efficacy and application: kV radiation is typically used for superficial treatments due to its limited penetration depth, while MV radiation penetrates deeper into tissues and is suitable for treating deeper-seated joints or larger affected areas. However, studies directly comparing the analgesic efficacy of kV and MV radiation specifically for a certain type of MSD are limited, and historically, both modalities have been shown to offer good symptomatic relief and improve joint function in roughly 60–80% of treated patients in clinical practice [4].

Germany is considered one of the leading countries in this field and has a long tradition of treating benign painful MSD with LDRT [4]. The treatment guideline published by the German Society of Radiation Oncology (DEGRO) reports a moderate evidence level, level B, for selection of the optimal treatment unit (kV vs. MV) because evidence levels (according to evidence-based medicine) cannot yet be applied for physical radiation parameters [5].

Novel conservative treatment approaches for painful MSD continue to attract research interest, particularly in the development of new therapeutic devices for LDRT. Many researchers are interested in determining which radiation source should be used for future device development for broad spectrum and easy use. In order to provide a comprehensive reference by synthesizing existing results, we have planned this systematic review and meta-analysis to compare the efficacy of kV and MV LDRT for treating painful MSD.

## Methods

### Search strategy

This meta-analysis was carried out according to a prespecified registered protocol (PROSPERO ID: CRD42023408374 at https://www.crd.york.ac.uk/prospero). In accordance with the Preferred Reporting Items for Systematic Reviews and Meta-Analyses (PRISMA) guidelines, we conducted a systematic literature search in PubMed, Embase, and the Cochrane Library databases to identify relevant studies. The PRISMA checklist is presented in Supplementary Table 1. The main search terms included “arthritis,” “joint disorder,” “radiotherapy” or “low-dose radiotherapy,” and “pain.” The detailed search strategies for each database are provided in Supplementary Table 2. We did not impose any language limitations. In cases where studies potentially had overlapping patient cohorts, we prioritized those with a larger number of patients or more recent publication dates.

### Inclusion and exclusion criteria

**Fig. 1:**
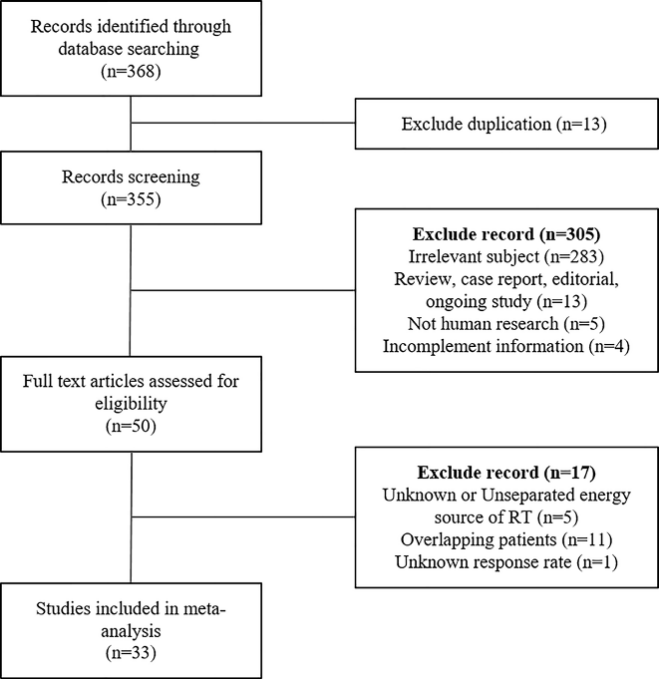
Flowchart of the study selection process

The inclusion criteria for this meta-analysis permitted inclusion of both prospective and retrospective studies. These studies were required to evaluate the pain response following LDRT with a known energy level, and each comparison arm had to have a minimum of 10 patients. Conversely, the exclusion criteria encompassed the article types of reviews, meta-analyses, case reports, editorials, conference abstracts, and ongoing clinical trials. Additionally, only human research was considered, with animal studies and experimental research excluded. Irrelevant subjects, such as pigmented villonodular synovitis (PVNS), heterotopic ossification, and Dupuytren’s contracture, were also excluded. Furthermore, studies lacking information about the energy source used for LDRT as well as those with incomplete information were excluded. The detailed study selection process is shown in Fig. 1.

### Data extraction

Data extraction was independently conducted by two authors (A.K. and J.K.). The data collected included study characteristics (publication year, author, institution, period, and study type), number of patients, LDRT details (dose/fraction, device/energy), follow-up duration, pain evaluation methods, and response outcomes at various timepoints. The primary outcome of interest was the overall response rate (ORR) of pain, and the secondary outcome of interest was the complete response rate (CRR) of pain. The incidences and types of adverse events were also collected and subjectively reviewed. Any discrepancies between the reviewers regarding the extraction of data were resolved through discussion.

The risk of bias was assessed using the Newcastle–Ottawa Scale for non-randomized studies [6] and a revised Cochrane risk of bias tool for randomized trials (RoB2 tool) [7]. Two authors (A.K. and J.K.) independently evaluated the quality of the included studies, and any discrepancies were resolved through discussion between authors.

### Statistical analysis

The primary endpoint was the pooled ORR for each energy level (kV vs. MV). Response rates with 95% confidence intervals (CI) were used as summary statistics. Pooled rates were calculated using the random-effects model. The levels of heterogeneity between studies were evaluated using the Cochrane Q test and *I*^*2*^ statistics. We defined the composite result of the response rate within 6 months as the short-term response rate and the composite result of the response rate after 12 months as the long-term response rate. Planned exploratory subgroup analyses (by treatment site, study period, study design, study country, and dose per fraction) were performed to investigate the sources of heterogeneity across the studies. Also, publication bias was assessed using contour-enhanced funnel plots and Egger’s regression tests. The R 4.2.1 Statistical Package (https://www.r-project.org/) was used to perform all statistical analyses.

## Results

### Study selection and characteristics

Initially, a total of 368 records were identified through the database search. Following the exclusion of duplicates, 355 records were screened based on title and abstract and 305 of these were excluded because they did not meet the selection criteria. Fifty records were shortlisted for full-text review, of which 17 records were further excluded, as detailed in Fig. 1. Consequently, 33 studies involving 12,143 patients meeting all the criteria were included in the following meta-analysis [8–40].

The characteristics of the included studies are detailed in Table 1. There were 9 prospective and 24 retrospective studies, all conducted within Europe. Most papers were published in Germany, and there were many different types of MSD involving various joints (knee, hand, foot, shoulder, hip, ankle, etc.). Unfortunately, none of the included studies compared response rates between kV and MV within a single study because such studies did not meet the selection criteria. This was due to reasons such as the timepoint at which the pain response rate was obtained not being specific or patient groups treated with kV and MV being reported together. In the case of papers reporting short- and long-term data separately, a pooled analysis was performed for each timepoint, even if the patient group was the same. A total of 12 studies used kV energy ranging from 120 to 250 kV, while 21 studies used MV energy ranging from 6 MV to 15 MV. In the majority of these studies, LDRT was administered at a dose of either 0.5 Gy or 1.0 Gy per fraction up to a total of 4–12 fractions.

**Table 1:** Summary characteristics of included studies

No	Study	Study period	Country	Study design	Joints	Total dose (dose per fraction)	Energy
1	Van den Ende et al. (2019) [8]	2015–2017	Netherlands	P	Knee (27), hand (28)	6 Gy (1.0 Gy)	M
2	Mahler et al. (2019) [9]	2015–2017	Netherlands	P	Knee (27)	6 Gy (1.0 Gy)	M
3	Minten et al. (2018) [10]	2016–2017	Netherlands	P	Hand (28)	6 Gy (1.0 Gy)	M
4	Ott et al. (2014) [11]	2006–2010	Germany	P	Foot (457)	6 Gy (1.0 Gy) (52.5%) 6 Gy (0.5 Gy) (47.5%)	K
5	Ott et al. (2013) [12]	2006–2010	Germany	P	Foot (457)	6 Gy (1.0 Gy) (52.5%) 6 Gy (0.5 Gy) (47.5%)	K
6	Hautmann et al. (2020) [47]	NA	Germany	R	Knee (102), hand (90), shoulder (36), ankle (29), hip (13), others (25)	6 Gy (1.0 Gy) (77.6%) 3 Gy (0.5 Gy) (19.0%) Others (3.4%)	M
7	Hautmann et al. (2020) [14]	2006–2018	Germany	R	Ankle (44), foot (22)	3 Gy (0.5 Gy) (60.6%) 6 Gy (1.0 Gy) (36.4%) 5 Gy (1.0 Gy) (3.0%)	M
8	Hautmann et al. (2019) [13]	2006–2017	Germany	R	Elbow (138)	6 Gy (1.0 Gy) (89.1%) 3 Gy (0.5 Gy) (9.4%) Others (1.5%)	M
9	Ayanaci et al. (2021) [16]	2013–2017	Turkey	R	Foot (67)	6 Gy (1.0 Gy)	M
10	Rogers et al. (2020) [17]	2013–2015	Switzerland	P	Hand (59), foot (54), elbow (44)	4 Gy (0.5 Gy) (If non-CR, up to 8 Gy)	K
11	Alvarez et al. (2019) [18]	2015–2018	Spain	P	Hand (51), hip (37), knee (33), shoulder (16), ankle (10), foot (8), elbow (4), others (25)	6 Gy (1.0 Gy) (If re-RT, repeat treatment)	M
12	Badakhsi et al. (2014) [19]	2002–2008	Germany	R	Foot (171)	3 Gy (0.5 Gy)	K
13	Weissmann et al. (2021) [20]	2004–2019	Germany	R	Foot (140), ankle (56)	3 Gy (0.5 Gy) (90.0%) 6 Gy (1.0 Gy) (10.0%)	K
14	Hermann et al. (2021) [21]	2016–2018	Germany	P	Hand (25)	3 Gy (0.5 Gy)	M
15	Niewald et al. (2022) [22]	NA	Germany	P	Hand (158), Knee (63)	3 Gy (0.5 Gy) (50.0%) 0.3 Gy (0.05 Gy) (50.0%)	M
16	Alvarez et al. (2022) [23]	2015–2021	Spain	R	Hand (100)	6 Gy (1.0 Gy) (84.0%) 3 Gy (0.5 Gy) (16.0%)	M
17	Rühle et al. (2021) [24]	2008–2020	Germany	R	Knee (419), hand (363), foot (219), shoulder (147), hip (33), others (4)	6 Gy (1.0 Gy) (77.3%) 3 Gy (0.5 Gy) (21.7%) Others (1.0%)	M
18	Rudat et al. (2021) [25]	2009–2020	Germany	R	Foot (864)	3 Gy (0.5 Gy) (90.9%) 6 Gy (1.0 Gy) (9.1%)	M
19	Donaubauer et al. (2020) [26]	2004–2019	Germany	R	Hand (483)	3 Gy (0.5 Gy) (95.45%) 6 Gy (1.0 Gy) (4.55%)	K
20	Juniku et al. (2019) [27]	2014–2015	Germany	R	Foot (194), shoulder (135), hand (95), hip (84), elbow (60), knee (30)	5 Gy (0.5 Gy) (94.3%) 3 Gy (0.5 Gy) (5.7%)	M
21	Kaltenborn et al. (2017) [28]	2007–2015	Germany	R	Hip (74)	6 Gy (1.0 Gy) (60.8%) 6 Gy (0.06 Gy) (39.2%)	M
22	Kaltenborn et al. (2016) [29]	2007–2013	Germany	R	Hand (101)	6 Gy (1.0 Gy)	M
23	Leszek et al. (2015) [30]	NA	Poland	R	Elbow (50)	6 Gy (1.0 Gy)	M
24	Hermann et al. (2013) [31]	2007–2009	Germany	R	Foot (285)	6 Gy (1.0 Gy) (84.6%) 3 Gy (0.5 Gy) (15.4%)	M
25	Hajtmanova et al. (2010) [32]	2007–2007	Slovakia	R	Foot (323)	4 Gy (1.0 Gy)	K
26	Adamietz et al. (2010) [33]	1999–2002	Germany	R	Shoulder (115)	6 Gy (0.5 Gy)	K
27	Keilholz et al. (1998) [34]	1984–1994	Germany	R	Knee (49), shoulder (27), hand (20)	6 Gy (0.5 Gy) (79.0%) 12 Gy (1.0 Gy) (21.0%)	K
28	Seegenschmiedt et al. (1997) [35]	1986–1991	Germany	R	Elbow (93)	6 Gy (1.0 Gy)	K
29	Heyd et al. (1997) [36]	NA	Germany	R	Elbow (45)	6 Gy (1.0 Gy)	M
30	Schafer et al. (1996) [37]	1985–1991	Germany	R	Shoulder (42), elbow (30), foot (18)	2–4 Gy (0.5–1.0 Gy)	M
31	Keilholz et al. (1995) [38]	1987–1991	Germany	R	Shoulder (89)	6 Gy (0.5 Gy)	K
32	Sautter-Bihl et al. (1993) [39]	1980–1991	Germany	R	Shoulder (74), knee (42), foot (24), elbow (15), others (26)	2.5–6 Gy (0.5 or 1.0 Gy)	M
33	Hess et al. (1988) [40]	1965–1985	Germany	R	Shoulder (164)	(0.3–0.5 Gy) (4–6 fractions)	K

*P* prospective, *R* retrospective, *M* megavoltage, *K* kilovoltage, *NA* not available, *re-RT* re-irradiation, *CR* complete response

*The patient cohorts in [9] and [10] are included in [8]. Reference [8] provides long-term follow-up data, whereas [9] and [10] provide short-term follow-up data

**The patient cohorts in [11] and [12] are the same. Reference [11] provides long-term follow-up data, whereas [12] provides short-term follow-up data

Each study had used various scales to assess the response rate of site-specific pain such as a numerical rating scale (NRS), a 10-point visual analogue scale (VAS), a comprehensive pain score (CPS), the von Pannewitz score (VPS), or the OMERACT-OARSI (Outcome Measures in Rheumatology-Osteoarthritis Research Society International) criteria for reporting overall responders. Pain response rates were inconsistently reported and had to be combined numerically across various pain scales. Despite this, the response was defined in each study based on clinically meaningful pain improvement, allowing the results to be seen as a reasonable estimate of what each modality can achieve clinically. Table 2 shows a detailed summary of pain response rates in the included studies.

**Table 2:** Summary of pain response rates in included studies

No	Study	Short-term		Long-term		Scale
		ORR	CRR	ORR	CRR	
1	Van den Ende et al. (2019) [8]	–	–	21/51 (41.2%)	–	OMERACT-OARSI responder
2	Mahler et al. (2019) [9]	12/27 (44.4%)	–	–	–	OMERACT-OARSI responder
3	Minten et al. (2018) [10]	8/28 (28.6%)	–	–	–	OMERACT-OARSI responder
4	Ott et al. (2014) [11]	–	–	285/300 (95%)	147/300 (49%)	CPS, VAS
5	Ott et al. (2013) [12]	187/212 (88.2%)	42/212 (19.8%)	–	–	CPS, VAS
6	Hautmann et al. (2020) [47]	178/271 (65.7%)	81/256 (31.6%)	137/216 (63.4%)	73/216 (33.8%)	NRS
7	Hautmann et al. (2020) [14]	36/48 (75%)	9/48 (18.8%)	35/46 (76.1%)	9/46 (19.6%)	NRS
8	Hautmann et al. (2019) [13]	–	65/125 (52%)	–	73/113 (64.6%)	NRS
9	AYNACI et al. (2021) [16]	48/67 (71.6%)	28/67 (41.8%)	–	–	VPS
10	Rogers et al. (2020) [17]	–	–	–	110/151 (72.8%)	VAS
11	Alvarez et al. (2019) [18]	90/184 (48.9%)	22/184 (12%)	57/89 (64%)	7/89 (7.9%)	VPS
12	Badakhsi et al. (2014) [19]	115/171 (67.3%)	–	–	–	Patient’s reported subjective response
13	Weissmann et al. (2021) [20]	96/196 (49%)	71/196 (36.2%)	–	–	Patient’s reported subjective response
14	Hermann et al. (2021) [21]	21/25 (84%)	1/25 (4%)	12/16 (75%)	3/16 (18.8%)	VPS
15	Niewald et al. (2022) [22]	130/220 (59.1%)	90/220 (40.9%)	–	–	VAS
16	Alvarez et al. (2022) [23]	–	–	63/100 (63%)	13/100 (13%)	VPS
17	Rühle et al. (2021) [24]	711/1185 (60%)	18/1185 (1.5%)	–	–	VPS
18	Rudat et al. (2021) [25]	372/582 (63.9%)	149/318 (46.9%)	216/318 (67.9%)	116/236 (49.2%)	VAS
19	Donaubauer et al. (2020) [26]	173/483 (35.8%)	–	–	–	Patient’s reported subjective response
20	Juniku et al. (2019) [27]	119/339 (35.1%)	–	373/598 (62.4%)	–	VAS
21	Kaltenborn et al. (2017) [28]	53/74 (71.6%)	17/52 (32.7%)	34/47 (72.3%)	24/47 (51.1%)	Patient’s reported subjective response
22	Kaltenborn et al. (2016) [29]	71/101 (70.3%)	9/65 (13.8%)	19/27 (70.4%)	7/27 (25.9%)	Patient’s reported subjective response
23	Leszek et al. (2015) [30]	23/28 (82.1%)	10/28 (35.7%)	21/24 (87.5%)	11/24 (45.8%)	Patient questionnaires
24	Hermann et al. (2013) [31]	–	–	208/252 (82.5%)	107/252 (42.5%)	Patient’s reported subjective response
25	Hajtmanova et al. (2010) [32]	180/323 (55.7%)	–	–	–	NA
26	Adamietz et al. (2010) [33]	–	–	94/115 (81.7%)	–	Orthopedic Constant Score
27	Keilholz et al. (1998) [34]	58/96 (60.4%)	35/96 (36.5%)	–	–	VPS
28	Seegenschmiedt et al. (1997) [35]	–	–	69/93 (74.2%)	50/93 (53.8%)	Morrey score
29	Heyd et al. (1997) [36]	31/45 (68.9%)	7/45 (15.6%)	–	–	Patient’s reported subjective response
30	Schafer et al. (1996) [37]	42/90 (46.7%)	–	68/90 (75.6%)	–	VPS
31	Keilholz et al. (1995) [38]	–	–	72/89 (80.9%)	44/89 (49.4%)	Patient’s objective, subjective symptoms
32	Sautter-Bihl et al. (1993) [39]	58/77 (75.3%)	29/77 (37.7%)	–	–	Patient’s reported subjective response
33	Hess et al. (1988) [40]	126/164 (76.8%)	–	–	–	Patient’s reported subjective response

*ORR* overall response rate, *CRR* complete response rate, *NRS* numeral rating scale, *VPS* von Pannewitz score, *VAS* visual analog scale, *CPS* comprehensive pain score, *OMERACT-OARSI* Outcome Measures in Rheumatology-Osteoarthritis Research Society International

### Pooled pain response rates by energy level

**Fig. 2:**
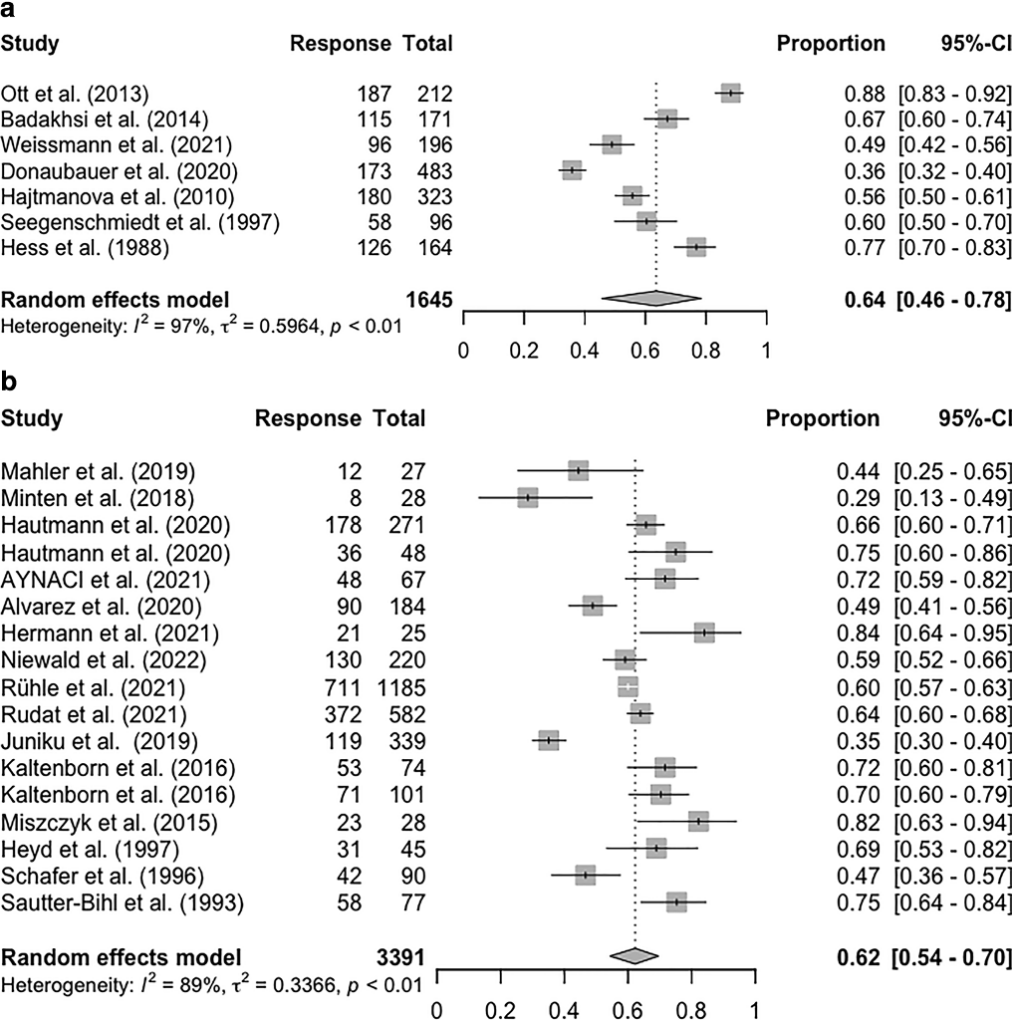
Forest plot of overall response rate in short-term follow-up. **a** kV and **b** MV. *CI *confidence interval

**Fig. 3:**
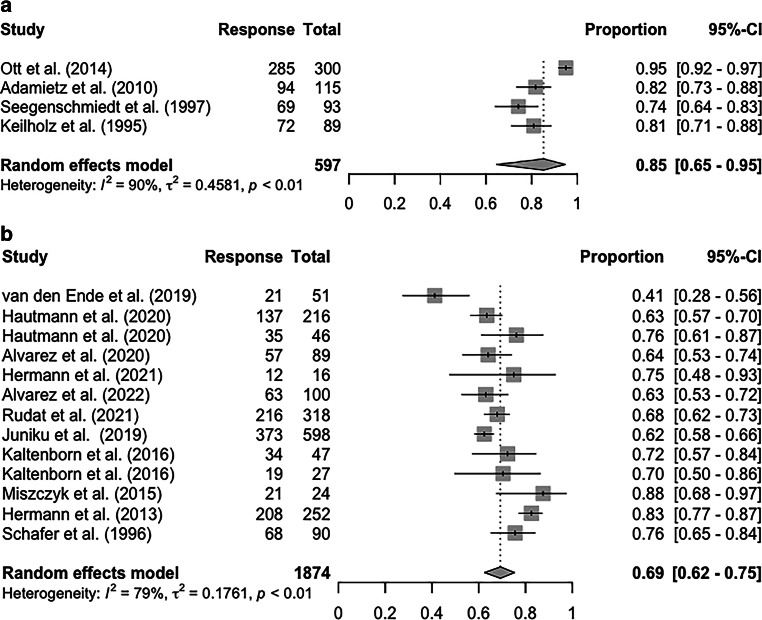
Forest plot of overall response rate in long-term follow-up. **a** kV and **b** MV.* CI *confidence interval

Primary forest plots are shown in Figs. 2 and 3. In the short-term follow-up data (up to 6 months after RT), the pooled ORR for kV and MV were 64% (95% CI 46–78%) and 62% (95% CI 54–70%), respectively (Fig. 2). However, inter-study heterogeneity was very high: *I*^*2*^ was 97% for kV and 89% for MV, respectively. The pooled short-term CRR for kV and MV, based on data from 504 and 2695 patients, were 30% (90% CI 14–54%) and 23% (95% CI 13–37%), respectively (Supplementary Fig. 1). As a result, no difference in short-term pain response was observed between the two energy levels.

In the long-term follow-up data (at least 12 months after RT), the pooled ORR was 85% (95% CI 65–95%) for kV (4 studies) and 69% (95% CI 62–75%) for MV (12 studies; Fig. 3). Again, very high heterogeneity was noted. The pooled long-term CRR for kV and MV, based on data from 633 and 1166 patients, were 57% (95% CI 40–72%) and 32% (95% CI 20–46%), respectively (Supplementary Fig. 2). Regardless of energy level, the composite long-term response rate was observed to be numerically higher than the short-term response rate.

**Fig. 4:**
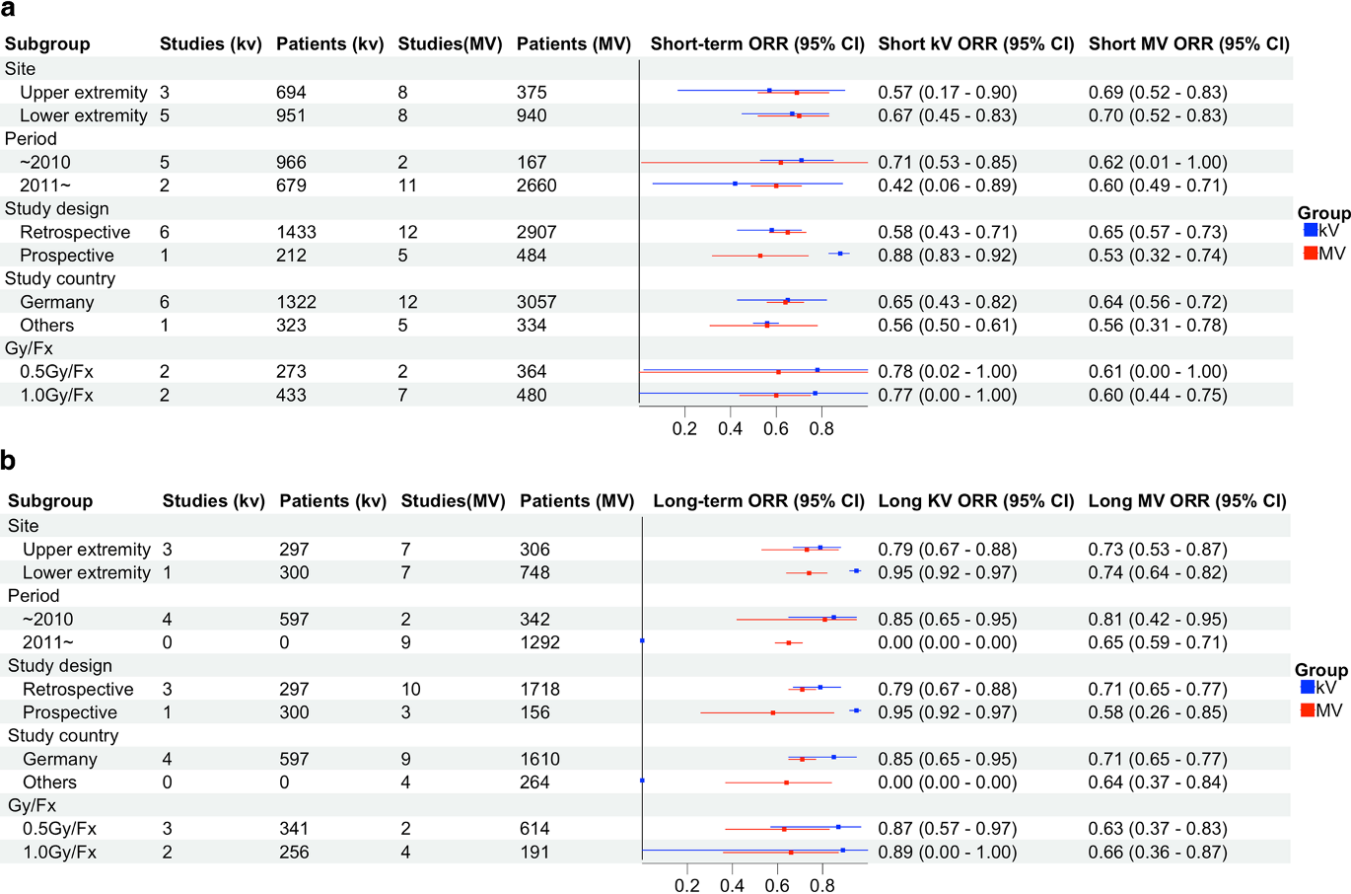
Subgroup analysis of overall response rate: **a** short-term follow-up and **b** long-term follow-up in kV vs. MV. *Fx *fraction, *CI *confidence interval, *ORR* overall response rate

In the exploratory subgroup analysis (Fig. 4), there were no significant differences in ORR by energy level stratified by treatment sites (upper extremity: shoulder, elbow, hand vs. lower extremity: hip, knee, ankle, and foot). In the short-term follow-up, the pooled ORR for the upper extremities with kV and MV were 66% (95% CI 32–89%) and 69% (95% CI 55–80%), respectively, while for the lower extremities they were 71% (95% CI 45–88%) and 75% (95% CI 57–88%), respectively. In the long-term follow-up, the pooled ORR for the upper extremities with kV and MV were 79% (95% CI 67–88%) and 82% (95% CI 67–91%), respectively. However, there was only one study on orthovoltage in the lower extremities, thus limiting the evaluation of response rates. Likewise, there were divergent response rates according to subgroups (stratified by study period, study design, country, and dose per fraction), but these results should be interpreted cautiously due to the small number of studies available for analysis. In the short-term follow-up, retrospective studies showed a slightly higher ORR for MV, while prospective studies showed a higher ORR for kV. From a regional perspective, the German studies demonstrated a higher ORR than others, but there was no significant difference in ORR between the two energies, with short-term response rates of 65% (95% CI 43–82%) for kV and 67% (95% CI 61–72%) for MV. Regarding dose per fraction (0.5 Gy vs. 1.0 Gy), both short- and long-term follow-up data demonstrated comparable ORR between the two energies.

### Incidences and types of adverse events

A few included studies mentioned acute, clinically insignificant nail reactions or fatigue during LDRT. No secondary malignancy was reported in these studies. Overall, side effects were insignificant, which corresponded to the reported absence of chronic or acute adverse effects in the literature [1, 3].

### Assessment for risk of bias and publication bias

A summary of the risk of bias assessment is provided in Supplementary Tables 3 and 4. The non-randomized studies generally demonstrated high quality in terms of selection and outcome assessment, despite the inherent limitation in comparability due to their single-arm design. The randomized controlled trials included in the analysis were mostly of high methodological quality, with a low risk of bias in most studies. Only a few studies exhibited some concerns, primarily related to the randomization process and deviations from the intended interventions.

The Egger’s test for the short-term kV group revealed statistically significant publication bias (*p* = 0.015), suggesting a potential for selective publication of studies with favorable outcomes. Conversely, the short-term MV group, as well as the long-term MV and kV groups, did not show statistically significant signs of publication bias (Supplementary Fig. 3.a–d). These findings imply a relatively equitable distribution of published studies, indicating that both significant and non-significant results have been fairly reported.

## Discussion

The optimal modality for applying LDRT is a subject of controversy. To the best of our knowledge, this study is the first meta-analysis to focus on comparing the effectiveness of LDRT by energy levels. In this systematic review/meta-analysis of 33 studies, we did not observe significant differences in pain response rates according to energy levels. Both kV and MV radiation can be effectively used for treating painful MSD according to our results. The choice between kV and MV depends on various factors, including the depth of the affected joint, the size of the treatment area, and the desired balance between efficacy and sparing normal tissues. kV radiation therapy may be limited by physical factors, while the use of MV radiation therapy may be limited by socioeconomic factors, requiring more specialized equipment and expertise, which may limit its availability in certain healthcare settings. The selection of the appropriate modality should be based on individual patient characteristics and treatment goals. Treatment decisions should be made in consultation with a radiation oncologist, considering the specific needs and circumstances of each patient.

However, our study has many limitations. Firstly, the heterogeneity of the included MSD is large. When limiting the analysis to specific diseases, the number of studies available for synthesis was insufficient, necessitating inclusion of a broad range of MSD. According to the LDRT consensus on MSD to date, a similar treatment scheme is recommended for all, mostly about six treatments with 0.5–1 Gy per fraction, which does not differ significantly depending on the causative disease. Therefore, our results, which analyze various MSD together, could be useful and align with previous systematic reviews [4]. Secondly, rather than comparing and reporting response rates according to energy level within each study, the results were aggregated at the study level. This approach presents a limitation as it involves comparing different patient groups. Additionally, because pain response rates were not reported on a unified basis, there was also an important pitfall that the response rates reported by various pain scales had to be numerically pooled. Despite this, each study defined the response based on a clinically meaningful pain improvement, allowing the results to approximate the clinical outcomes achievable with each modality (kV and MV). It is also important to note that the statistical power to detect publication bias is limited in groups with a small number of studies (i.e., *k* < 10, where *k* denotes the number of studies), such as in the short- and long-term kV groups. This limitation necessitates cautious interpretation of these findings, as the true extent of publication bias may not be fully detectable with the available data. Future research should include a larger number of studies to enhance the robustness of the findings and provide a more comprehensive evaluation of publication bias across different treatment modalities and timeframes. Lastly, since long-term follow-up data are more likely to have been interfered with by other pain control methods, the contribution of LDRT to the observed effects may be lower compared to short-term data. This potential confounding factor should be considered when interpreting the long-term efficacy of LDRT.

A German national patterns of care study published in 2004 for LDRT in plantar fasciitis showed that LDRT was delivered with kV units in 38.2% and with MV linear accelerators in 53.7% [41]. As expected, treatment success rates were not significantly different, at 81% with kV and 78% with MV. Another study in 2018 outlined the impact of different radiation treatment units on the rate of good response observed in patients with gonarthrosis, trochanteric bursitis , and shoulder syndrome, upon both completion of RT and during follow-up assessments [42]. Among 86 patients with gonarthrosis treated with MV, 31.4% showed a good response at the end of RT while 32.8% maintained a good response during follow-up. On the other hand, the rates were slightly lower in the kV group, with 30.1% exhibiting a good response post-RT and 21.6% maintaining it during follow-up, but there was no significant difference between the two treatment units (*p* = 0.882 for completion of RT and *p* = 0.218 for follow-up). For patients with shoulder syndrome, among 106 patients treated with MV, 27.4% exhibited a good response post-RT, while 57.4% maintained it during follow-up. In the kV group comprising 56 patients, a slightly higher percentage (42.8%) showed a good response post-RT, and 67.8% maintained it during follow-up. Although only published as an abstract (and thus not included in this meta-analysis), Scholocker et al. reported that the pain relief after LDRT of calcaneal spur was not influenced by radiation quality (orthovoltage vs. high voltage) [43]. Similarly, Frohlich et al. stated that 6 MV showed better results than 175 kV in painful heel spurs; however, the difference did not reach statistical significance in their abstract at ESTRO 2004 [44]. Overall, little research has suggested that a certain type of energy level may be more effective in treating specific types of MSD; therefore, we need more evidence on this aspect.

LDRT has been shown to reduce inflammation and pain associated with MSD; however, so far, no clear biologic in vitro, in vivo, or clinical outcome data exist to support a specific radiation modality (kV vs. MV) or parameters (dose rate, treatment intervals, quality factor like linear energy transfer [LET]) for each disease, as referred to in DEGRO guidelines [5, 45]. Inflammatory responses, typically modulated via NF-κB, exhibited stronger activation in the majority of cell types when exposed to high-LET radiation; therefore from this perspective, low-LET radiation may be likely to be more advantageous in inducing anti-inflammatory effects. [46]. Hautmann et al. suggested that kV radiation may be superior for treating OA due to its higher absorption in adjacent tissues, potentially leading to enhanced anti-inflammatory effects [47]. Moreover, the clinical effectiveness of LDRT over sham irradiation has been questioned by recent small randomized trials using MV radiation for OA [9, 10]. Therefore, we require a more substantial biological justification for each MSD, including OA, rather than the generalized concept of uniform LDRT for all.

In conclusion, this meta-analysis suggests that the known effectiveness of LDRT in painful degenerative MSD may not depend on the level of energy used. Notably, the heterogeneous assessment methods for response rates resulted in inconsistent reporting among studies. A prospective investigation to prove a possible differential effect in terms of energy level is needed. Additional studies using standardized evaluation methods are warranted to establish consistency and enhance the comprehensiveness of research. Although it has not yet been reported, further research is also needed to explore treatment modality selection and/or LDRT parameters while considering cause-specific biology.

## Supplementary Information

Supplementary figure 1. Forest plot of complete response rate in short-term follow-up. (a) kV and (b) MV

Supplementary figure 2. Forest plot of complete response rate in long-term follow-up. (a) kV and (b) MV

Supplementary figure 3. Funnel plot for publication bias (a) short-term, kV, (b) short-term, MV, (c) long-term, kV and (d) long-term, MV

Supplementary table 1. The PRISMA checklist

Supplementary table 2. Detailed search strategy in each database

Supplementary table 3. Quality assessment of studies enrolled using Newcastle–Ottawa scores for non-randomized trials

Supplementary table 4. Quality assessment of studies enrolled using the risk-of-bias tool (RoB 2) for randomized trials
