# Supplementary figures and images for: Comparative effectiveness of kilo- and megavoltage energies in low-dose radiotherapy for painful degenerative musculoskeletal diseases: a systematic review and meta-analysis
Source: Strahlenther Onkol. 2024 Dec 4;201(5):483–94. doi: 10.1007/s00066-024-02329-0 (PMC12014772; doi:10.1007/s00066-024-02329-0)

Supplementary figure 1. Forest plot of complete response rate in short-term follow up. (a) kV and (b) MV


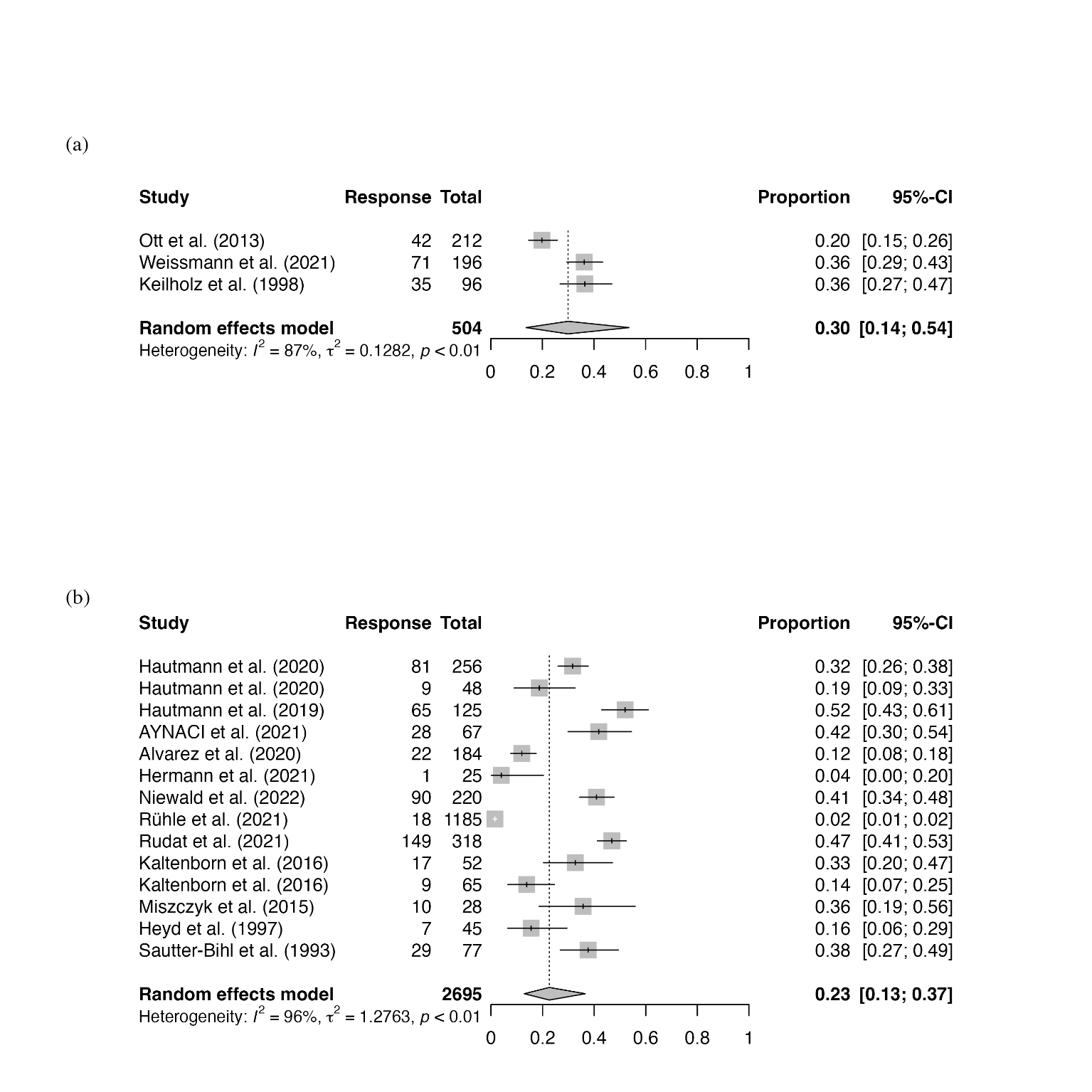

Supplement: Supplementary file 1 — Supplementary figure 1. Forest plot of complete response rate in short-term follow-up. (a) kV and (b) MV [file 66_2024_2329_MOESM1_ESM.docx]

Supplementary figure 2. Forest plot of complete response rate in long-term follow up. (a) kV and (b) MV


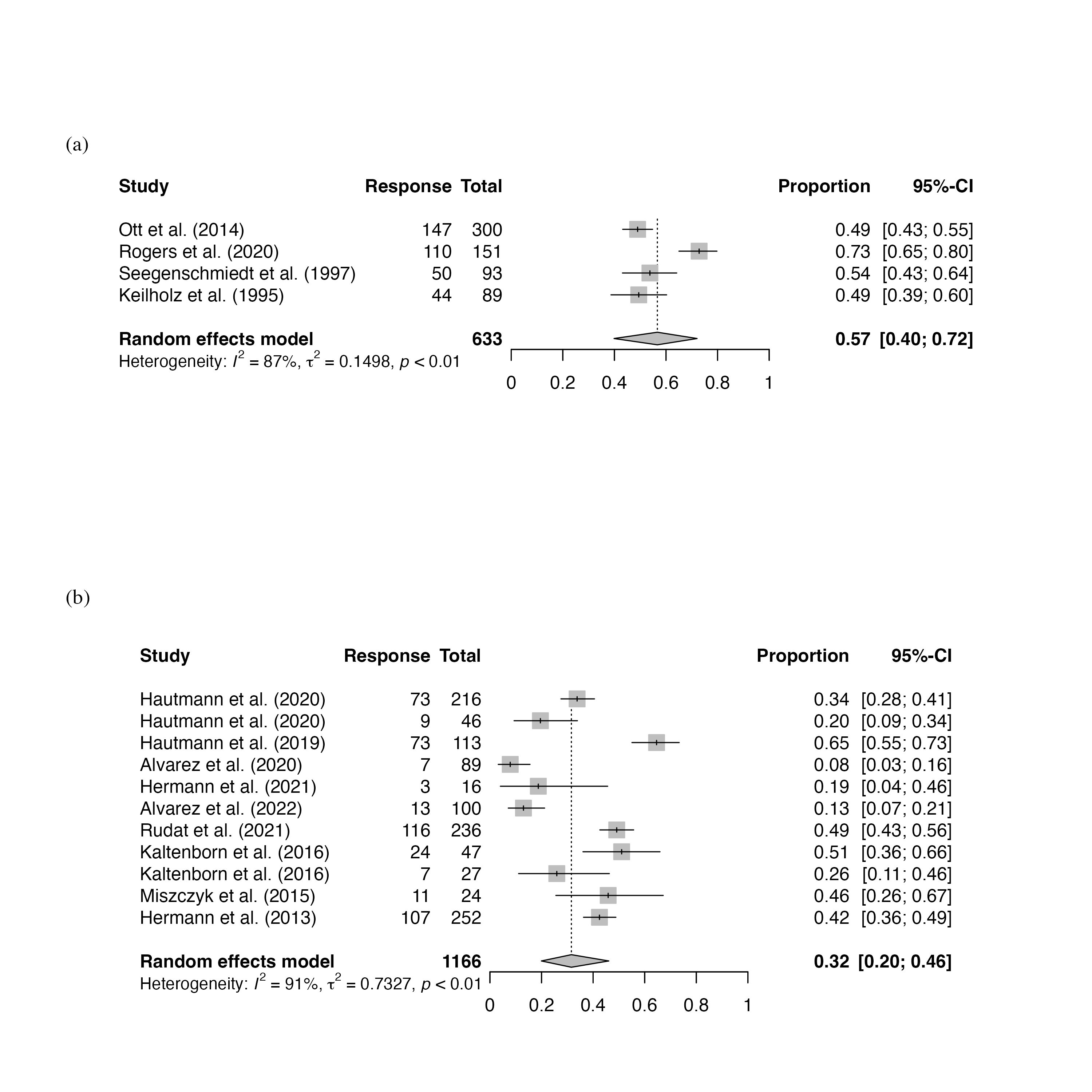

Supplement: Supplementary file 2 — Supplementary figure 2. Forest plot of complete response rate in long-term follow-up. (a) kV and (b) MV [file 66_2024_2329_MOESM2_ESM.docx]
