# Supplementary material for: Comparative effectiveness of kilo- and megavoltage energies in low-dose radiotherapy for painful degenerative musculoskeletal diseases: a systematic review and meta-analysis
Source: Strahlenther Onkol. 2024 Dec 4;201(5):483–94. doi: 10.1007/s00066-024-02329-0 (PMC12014772; doi:10.1007/s00066-024-02329-0)

Supplementary figure 3. Funnel plot for publication bias (a) short term, kV, (b) short-term, MV, (c) long-term, kV and (d) long-term, MV

(a)


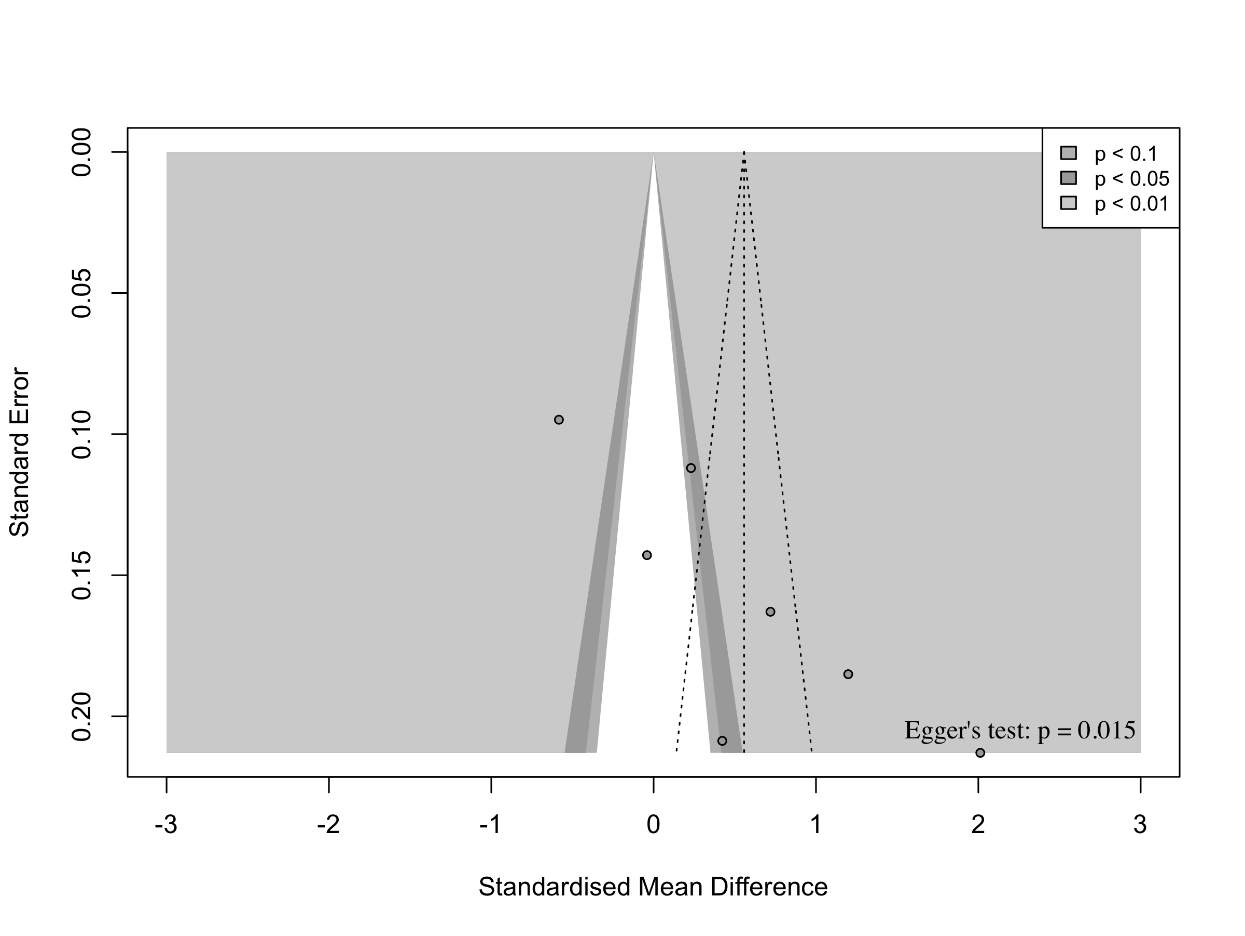


(b)


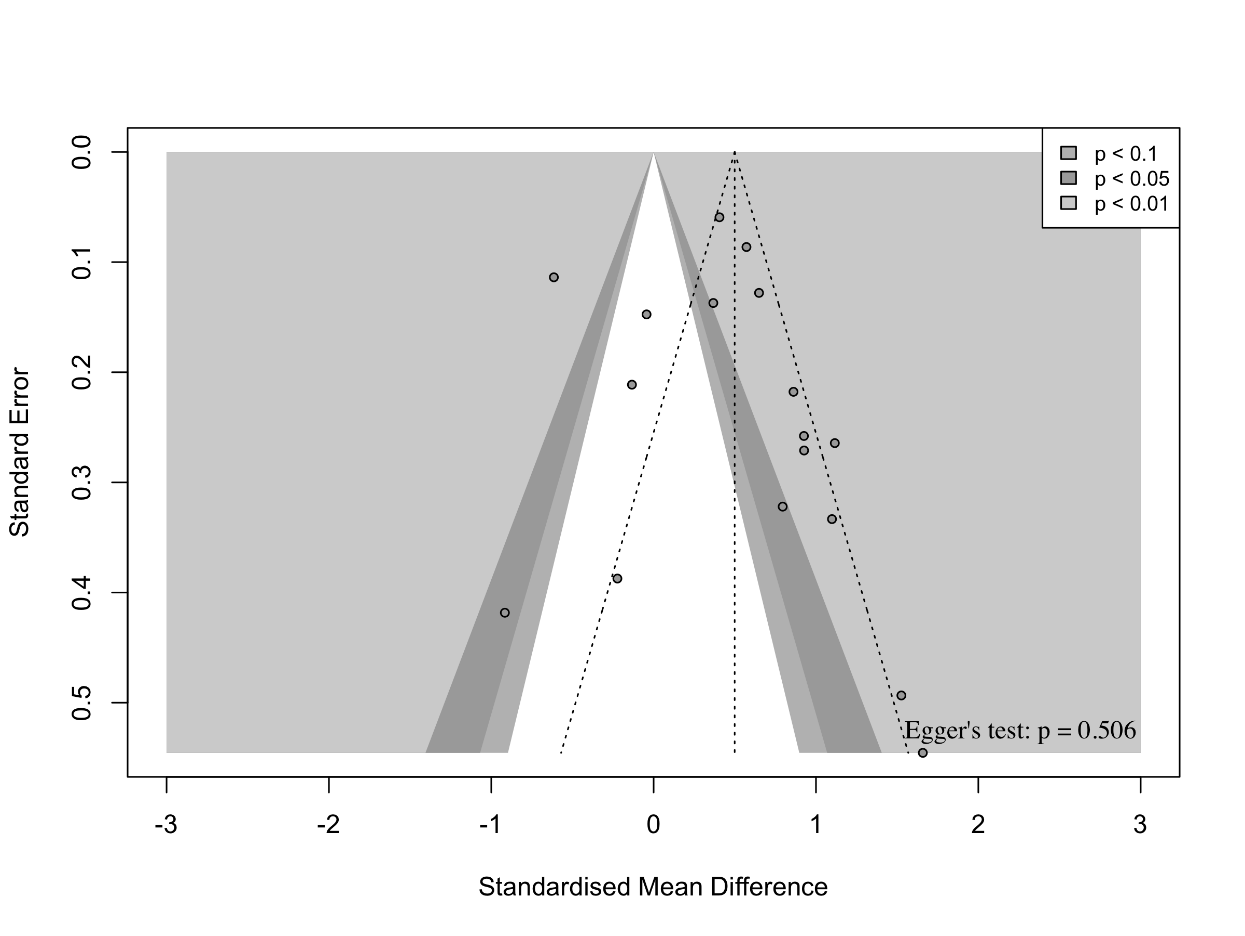


(c)


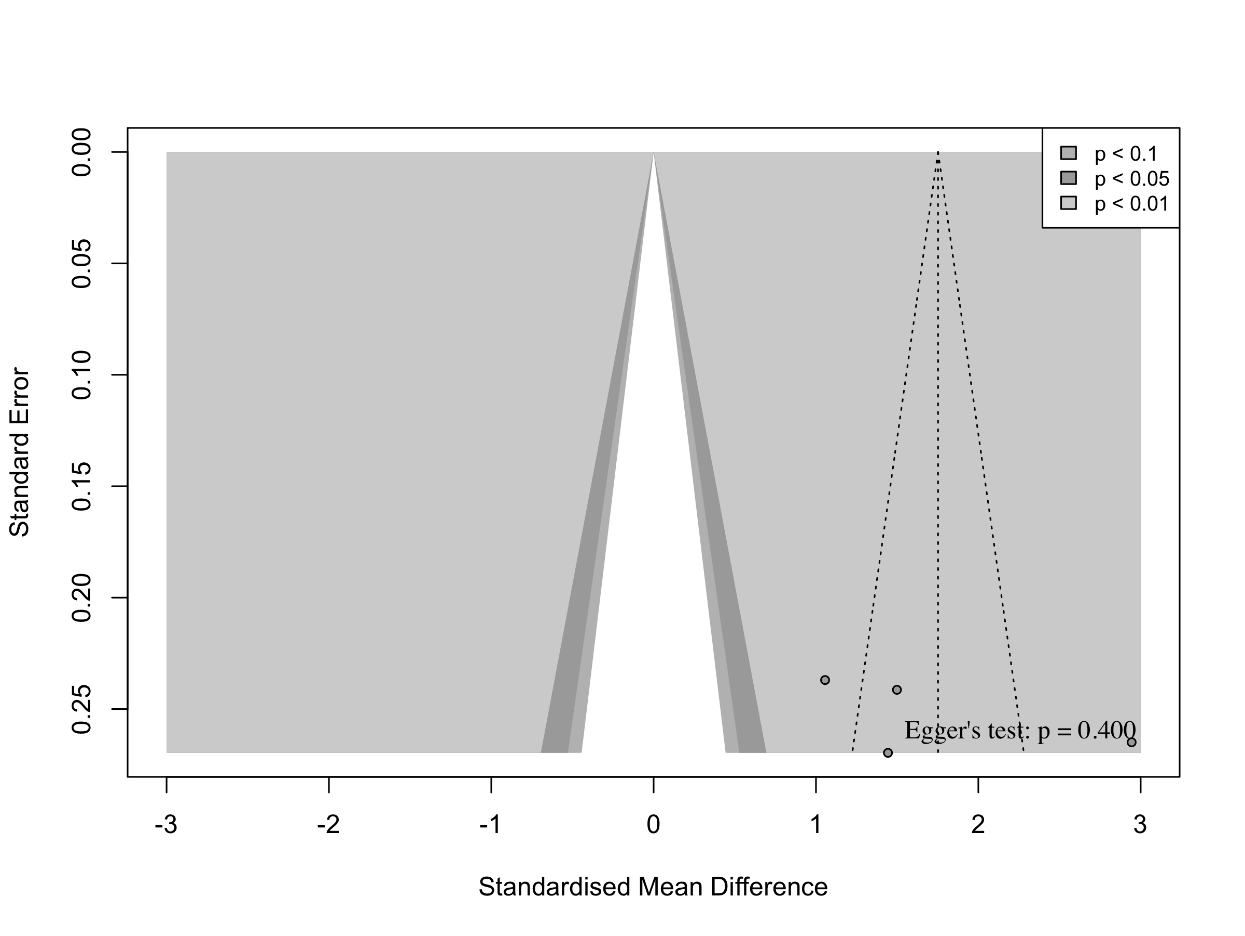


(d)


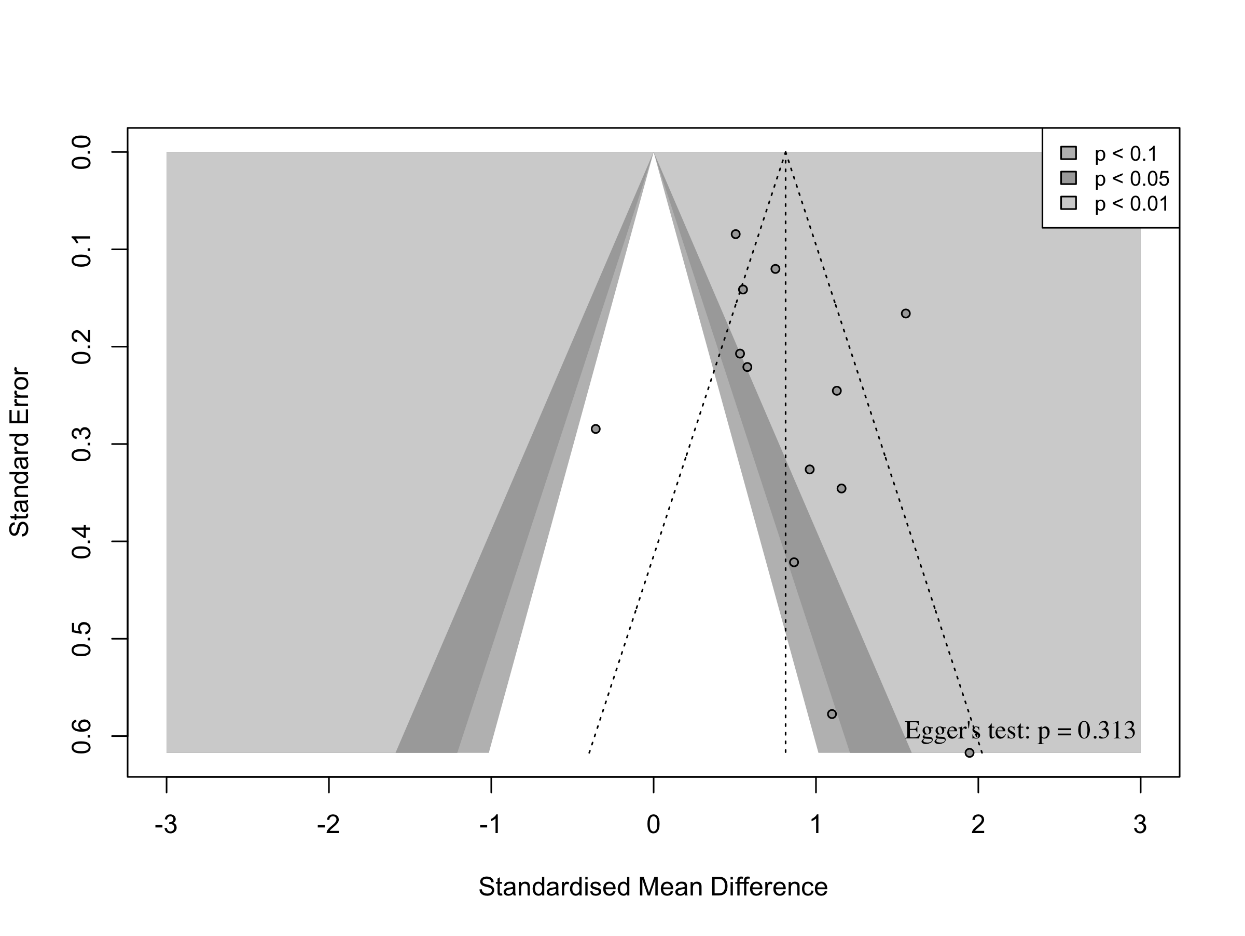

Supplement: Supplementary file 3 — Supplementary figure 3. Funnel plot for publication bias (a) short-term, kV, (b) short-term, MV, (c) long-term, kV and (d) long-term, MV [file 66_2024_2329_MOESM3_ESM.docx]
