# Supplementary material for: Comparative effectiveness of kilo- and megavoltage energies in low-dose radiotherapy for painful degenerative musculoskeletal diseases: a systematic review and meta-analysis
Source: Strahlenther Onkol. 2024 Dec 4;201(5):483–94. doi: 10.1007/s00066-024-02329-0 (PMC12014772; doi:10.1007/s00066-024-02329-0)
Supplement: Supplementary file 5 — Supplementary table 2. Detailed search strategy in each database [file 66_2024_2329_MOESM5_ESM.docx]

Supplementary table 2. Detailed search strategy in each database

**a:** Search strategy in PubMed

| # | Query |
| --- | --- |
| #1 | “arthritis”[mh] OR Joint disease[tiab] OR Joint disorder[tiab] OR osteoarthritis[tiab] OR osteoarticular degenerative[tiab] OR gonarthritis[tiab] OR gonarthrosis[tiab] OR arthrosis[tiab] OR benign painful skeletal disease[tiab] OR epicondylitis[tiab] OR bursitis trochanterica[tiab] OR calcaneodynia[tiab] OR epicondylopathia humeri[tiab] OR plantar fasciitis[tiab] OR painful musculoskeletal disease[tiab] OR shoulder tendinitis[tiab] OR bursitis[tiab] OR degenerative joint disease[tiab] OR polyarthritis[tiab] OR achillodynia[tiab] OR synovitis[tiab] |
| #2 | Pain[tiab] |
| #3 | “Radiotherapy”[mh] OR Radiotherapies[tiab] OR Radiation Therapy[tiab] OR Radiation Therapies[tiab] OR Radiation Treatment[tiab] OR low-dose radiotherapy[tiab] OR LDRT[tiab] OR low-dose radiation[tiab] OR low-dose radiation therapy[tiab] OR low-dose irradiation[tiab] OR x-ray irradiation[tiab] OR ionizing radiation[tiab] |
| #4 | NOT (Review[Publication Type]) |
| #5 | #1 AND #2 AND #3 AND #4 |

**b:** Search strategy in Embase

| # | Query |
| --- | --- |
| #1 | ‘arthritis’/exp OR ‘Joint disease':ab,ti OR ‘Joint disorder':ab,ti OR ‘osteoarthritis':ab,ti OR ‘osteoarticular degenerative':ab,ti OR ‘gonarthritis':ab,ti OR ‘gonarthrosis':ab,ti OR ‘arthrosis':ab,ti OR ‘benign painful skeletal disease':ab,ti OR ‘epicondylitis':ab,ti OR ‘bursitis trochanterica':ab,ti OR ‘calcaneodynia':ab,ti OR ‘epicondylopathia humeri':ab,ti OR ‘plantar fasciitis':ab,ti OR ‘painful musculoskeletal disease':ab,ti OR ‘shoulder tendinitis':ab,ti OR ‘bursitis':ab,ti OR ‘degenerative joint disease':ab,ti OR ‘polyarthritis':ab,ti OR ‘achillodynia':ab,ti OR ‘synovitis':ab,ti |
| #2 | ‘pain':ab,ti |
| #3 | ‘Radiotherapy’/exp OR ‘Radiotherapies':ab,ti OR ‘Radiation Therapy':ab,ti OR ‘Radiation Therapies':ab,ti OR ‘Radiation Treatment':ab,ti OR ‘low-dose radiotherapy':ab,ti OR ‘LDRT':ab,ti OR ‘low-dose radiation':ab,ti OR ‘low-dose radiation therapy':ab,ti OR ‘low-dose irradiation':ab,ti OR ‘x-ray irradiation':ab,ti OR ‘ionizing radiation':ab,ti |
| #4 | #1 AND #2 AND #3 |
| #5 | #4 AND ('article'/it OR 'article in press'/it) |
| #6 | #5 AND [embase]/lim NOT ([embase]/lim AND [medline]/lim) |

**c:** Search strategy in Cochrane Library

| # | Query |
| --- | --- |
| #1 | MeSH descriptor: [Arthritis] explode all trees |
| #2 | (Joint disease OR Joint disorder OR osteoarthritis OR osteoarticular degenerative OR gonarthritis OR gonarthrosis OR arthrosis OR benign painful skeletal disease OR epicondylitis OR bursitis trochanterica OR calcaneodynia OR epicondylopathia humeri OR plantar fasciitis OR painful musculoskeletal disease OR shoulder tendinitis OR bursitis OR degenerative joint disease OR polyarthritis OR achillodynia OR synovitis):ti,ab,kw |
| #3 | #1 OR #2 |
| #4 | (pain):ti,ab,kw |
| #5 | (Radiotherapy OR Radiotherapies OR Radiation Therapy OR Radiation Therapies OR Radiation Treatment OR low-dose radiotherapy OR LDRT OR low-dose radiation OR low-dose radiation therapy OR low-dose irradiation OR x-ray irradiation OR ionizing radiation):ti,ab,kw |
| #6 | #3 AND #4 AND #5 |
| #7 | "accession number" near pubmed |
| #8 | "accession number" near2 embase |
| #9 | #7 or #8 |
| #10 | #6 NOT #9 |
