# Supplementary material for: Comparative effectiveness of kilo- and megavoltage energies in low-dose radiotherapy for painful degenerative musculoskeletal diseases: a systematic review and meta-analysis
Source: Strahlenther Onkol. 2024 Dec 4;201(5):483–94. doi: 10.1007/s00066-024-02329-0 (PMC12014772; doi:10.1007/s00066-024-02329-0)
Supplement: Supplementary file 6 — Supplementary table 3. Quality assessment of studies enrolled using Newcastle–Ottawa scores for non-randomized trials [file 66_2024_2329_MOESM6_ESM.docx]

Supplementary table 3. Quality assessment of studies enrolled using Newcastle-Ottawa scores for non-randomized trials.

| Study | Selection | | | | Comparability | Outcome | | | Total |
| --- | --- | --- | --- | --- | --- | --- | --- | --- | --- |
|  | 1 | 2 | 3 | 4 | 5 | 6 | 7 | 8 |  |
| Hautmann et al. (2020) | * | NA | * | * | NA | 0 | * | * | 5/6 |
| Hautmann et al. (2020) | * | NA | * | * | NA | 0 | * | * | 5/6 |
| Hautmann et al. (2019) | * | NA | * | * | NA | 0 | * | * | 5/6 |
| AYNACI et al. (2021) | * | * | * | * | * | 0 | * | * | 7/9 |
| Rogers et al. (2020) | * | NA | * | * | NA | 0 | * | * | 5/6 |
| Alvarez et al. (2020) | * | NA | * | * | NA | 0 | * | * | 5/6 |
| Badakhsi et al. (2014) | * | NA | * | * | NA | 0 | * | * | 5/6 |
| Weissmann et al. (2021) | * | NA | * | * | NA | 0 | * | * | 5/6 |
| Hermann et al. (2021) | * | NA | * | * | NA | 0 | * | * | 5/6 |
| Alvarez et al. (2022) | * | NA | * | * | NA | 0 | * | * | 5/6 |
| Rühle et al. (2021) | * | NA | * | * | NA | 0 | * | * | 5/6 |
| Rudat et al. (2021) | * | NA | * | * | NA | 0 | * | * | 5/6 |
| Donaubauer et al. (2020) | * | NA | * | * | NA | 0 | * | * | 5/6 |
| Juniku et al. (2019) | * | NA | * | * | NA | 0 | * | * | 5/6 |
| Kaltenborn et al. (2016) | * | NA | * | * | NA | 0 | * | * | 5/6 |
| Kaltenborn et al. (2016) | * | NA | * | * | NA | 0 | * | * | 5/6 |
| Miszczyk et al. (2015) | * | NA | * | * | NA | 0 | * | * | 5/6 |
| Hermann et al. (2013) | * | NA | * | * | NA | 0 | * | * | 5/6 |
| Hajtmanova et al. (2010) | * | NA | * | * | NA | 0 | * | * | 5/6 |
| Adamietz et al. (2010) | * | NA | * | * | NA | 0 | * | * | 5/6 |
| Keilholz et al. (1998) | * | NA | * | * | NA | 0 | * | * | 4/6 |
| Seegenschmiedt et al. (1997) | * | NA | * | * | NA | 0 | * | * | 5/6 |
| Heyd et al. (1997) | * | NA | * | * | NA | 0 | * | * | 5/6 |
| Schafer et al. (1996) | * | NA | * |  | NA | 0 | * | * | 4/6 |
| Keilholz et al. (1995) | * | NA | * | * | NA | 0 | * | * | 5/6 |
| Sautter-Bihl et al. (1993) |  | NA | * |  | NA | 0 | * | * | 3/6 |
| Hess et al. (1988) | * | NA | * | * | NA | 0 | * | * | 5/6 |

*In single-arm study, items 2 and 5 were recorded as “NA (not applicable)”
