# Supplementary material for: Comparative effectiveness of kilo- and megavoltage energies in low-dose radiotherapy for painful degenerative musculoskeletal diseases: a systematic review and meta-analysis
Source: Strahlenther Onkol. 2024 Dec 4;201(5):483–94. doi: 10.1007/s00066-024-02329-0 (PMC12014772; doi:10.1007/s00066-024-02329-0)
Supplement: Supplementary file 7 — Supplementary table 4. Quality assessment of studies enrolled using the risk-of-bias tool (RoB 2) for randomized trials [file 66_2024_2329_MOESM7_ESM.docx]

Supplementary table 4. Quality assessment of studies enrolled using Risk-of-bias tool (RoB 2) for randomized trials

| Study | D1 | D2 | D3 | D4 | D5 | Total |
| --- | --- | --- | --- | --- | --- | --- |
| van den Ende et al. (2019) | Low | Low | Low | Low | Low | Low |
| Mahler et al. (2019) | Low | Low | Low | Low | Low | Low |
| Minten et al. (2018) | Low | Low | Low | Low | Low | Low |
| Ott et al. (2014) | Some concerns | Some concerns | Low | Low | Low | Some concerns |
| Ott et al. (2013) | Some concerns | Some concerns | Low | Low | Low | Some concerns |
| Niewald et al. (2022) | Low | Low | Low | Low | Low | Low |
